# Adult-Onset Hypopigmented Mycosis Fungoides: A Systematic Review of Clinicopathologic, Immunophenotypic, and Therapeutic Characteristics

**DOI:** 10.3390/cancers18020265

**Published:** 2026-01-15

**Authors:** Agnieszka Kimak-Pielas, Ewa Robak, Tadeusz Robak, Agnieszka Żebrowska

**Affiliations:** 1 Department of Dermatology and Venereology, Medical University of Lodz, 90-647 Łódź, Poland; ewa.robak@umed.lodz.pl (E.R.); agnieszka.zebrowska@umed.lodz.pl (A.Ż.); 2 Department of Dermatology and Venereology, Teaching Hospital No 2, 90-549 Łódź, Poland; 3 Department of Hematology, Medical University of Lodz, 91-738 Łódź, Poland; tadeusz.robak@umed.lodz.pl; 4 Department of General Hematology, Copernicus Memorial Hospital, 93-510 Łódź, Poland

**Keywords:** hypopigmented mycosis fungoides, hMF, Mycosis fungoides, cutaneous T cell lymphoma, CTCL, adult-onset

## Abstract

**Background/Objectives**: Hypopigmented mycosis fungoides (hMF) is a rare variant of mycosis fungoides (MF) often seen in younger patients and individuals with darker skin phototypes. The lesions develop as hypopigmented patches or plaques, and they are usually asymptomatic and respond well to topical treatment or phototherapy. **Methods**: We provide a systematic review on hMF with onset at or beyond 30 years of age, based on SCOPUS, PubMed, and Embase databases. A total of 13 original articles, totaling 34 patients, were included in this review. Evidence was limited to case reports and small series; PROSPERO registration is CRD420251181894. **Results**: The majority of cases did not progress beyond stage IB and commonly used treatment methods, including topical corticosteroids and phototherapy. In three patients, a progression of the disease occurred, and in two of them it was fetal. Among patients receiving phototherapy, PUVA therapy achieved complete remission more often than UVB (13 out of 17 cases vs. 8 out of 16 cases). Although recurrences occurred with both treatments, they were less frequent, and relapses took longer to develop in the PUVA group. **Conclusions**: In this cohort, PUVA appeared to be associated with higher complete response rates and longer remission duration than UVB. However, this advantage of PUVA is derived from low-level evidence and should be confirmed in prospective comparative studies.

## Simple Summary

Hypopigmented mycosis fungoides is a rare form of skin cancer that usually appears as light patches on the skin, most commonly in younger people or those with darker skin tones. Typically, it is not painful, not itchy, and can be treated with topical medications or light therapy. In this article, we reviewed published cases of this condition developing in people older than 30 years old, which are very rare. Most patients remained at an early stage and were successfully managed with phototherapy. Our findings may offer valuable insights into better diagnosis and treatment options for patients facing similar challenges.

## 1. Introduction

Mycosis fungoides (MF) is the most common type of primary cutaneous T-cell lymphoma (CTCL), typically affecting adults in their 40s and 50s. Lesions usually develop in sun-protected areas and may present as non-infiltrated patches and macules, infiltrated plaques, tumors, or erythroderma. The course of mycosis fungoides is typically indolent and chronic, with patients often remaining in the early stages for many years. However, once the disease reaches the tumor stage, the prognosis is significantly worse [1].

Apart from the classic Alibert–Bazin subtype, and three main subtypes (folliculotropic, pagetoid reticulosis, and granulomatous slack skin), various rare MF subtypes have been identified. Hypopigmented mycosis fungoides (hMF) most commonly affects dark-skinned individuals and is often described as a juvenile type of MF, typically presenting before the age of 30. Skin lesions are hypo- or achromic, usually non-infiltrated, and develop on the trunk, buttocks, and proximal parts of the limbs. They can vary in number and size and are typically asymptomatic [2]. In some cases, hypopigmented lesions may be accompanied by erythema, which is a more common pattern in the Caucasian population [3].

Diagnosis of hMF relies on histopathological examination and may require multiple skin samples. Alongside classic MF features, including epidermal and upper dermal lymphocytic infiltrates, the hypopigmented variant typically exhibits a predominance of CD8+ T-cells, which may contribute to its more indolent course. In doubtful cases, T-cell receptor (TCR) gene rearrangement analysis may be helpful in establishing the diagnosis [2,4,5,6,7].

HMF may mimic many conditions, and the most common misdiagnosis is vitiligo or leprosy in endemic regions [8,9,10,11]. The prognosis of hMF is generally excellent, with good responses to phototherapy or other skin-directed therapies. However, relapses are common. Although rare, a few lethal cases of hMF have been reported, so patients always require assessment of lymph nodes, blood, and systemic involvement [2,12,13].

Most of the available literature on hypopigmented mycosis fungoides focuses on pediatric, adolescent, or young adult populations and consists primarily of case reports and small case series. Data on adult-onset hMF and evidence-based treatment recommendations for this group remain limited. The aim of this review is to provide a comprehensive characterization of late-onset hMF, with a particular focus on treatment effectiveness.

## 2. Materials and Methods

This systematic review was conducted in accordance with the PRISMA guidelines and was prospectively registered in the PROSPERO database (ID: CRD420251181894). Research on adult-onset (30 years old or older) hypopigmented mycosis fungoides was independently conducted on 11 November 2025 by A.K.-P. and A.Ż. Both reviewers independently executed the search strategy across the selected electronic databases and assessed each study. Titles and abstracts of retrieved records were screened independently by both reviewers, and full-text articles were subsequently assessed for eligibility. Any disagreements between reviewers at any stage of the screening or selection process were resolved through discussion and consensus.

The search included terms (“Hypopigmented mycosis fungoides” [Title/Abstract] OR “hypopigmented MF” [Title/Abstract] OR “mycosis fungoides, hypopigmented” [MeSH Terms]) for PubMed, (‘hypopigmented mycosis fungoides’ OR ‘hypopigmented MF’) for Embase, and (TITLE-ABS (“hypopigmented mycosis fungoides”) OR TITLE-ABS (“hypopigmented MF”)) (hypopigmented [Title/Abstract] AND mycosis [Title/Abstract] AND fungoides [Title/Abstract]) OR (hypopigmented [Title/Abstract] AND MF [Title/Abstract]) for the Scopus database. To maximize sensitivity for identifying all publications related to hypopigmented mycosis fungoides, age-related terms (e.g., “adult,” “age of onset”) were deliberately not included in the search strings, as patient age and age of disease onset are frequently not reported in titles or abstracts. Instead, the adult-onset criterion was applied during title, abstract, and full-text screening. All retrieved records were manually assessed, and only studies meeting this criterion were included in the final analysis, ensuring comprehensive identification of the relevant literature while minimizing the risk of omission. The articles written in English were included, and in total, 540 potentially eligible articles were detected. An additional four relevant publications were identified by reviewing the reference lists of the selected articles.

**Figure 1:**
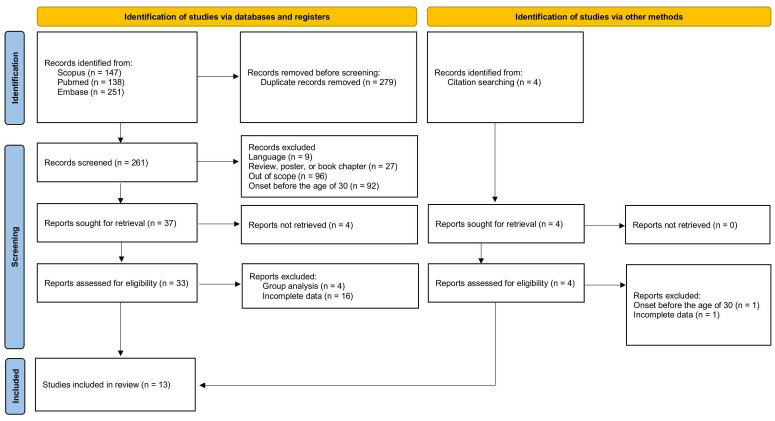
PRISMA flow chart. Group analysis—patient data reported collectively. Incomplete data—missing information on treatment, outcomes, or follow-up.

Based on the titles, abstracts, and full-text articles, after removing duplicates and studies that were irrelevant, addressed other topics, were written in languages other than English, or focused on individuals under 30 years of age at hMF onset, 33 original articles were identified. To compare treatment effectiveness, the review included only articles that included complete data, i.e., specified the treatment method, reported outcomes, and provided a defined follow-up period. Sixteen articles with incomplete information were therefore excluded. After removing studies that analyzed patient groups collectively rather than as individual cases, we ultimately included 13 articles totaling 34 cases of hMF onset occurring at 30 years of age or later, with specified treatment effectiveness, outcome, and follow-up period (Figure 1) [5,6,7,12,13,14,15,16,17,18,19,20,21]. The articles included in the review are summarized in Table 1.

## 3. Results

A total of 34 patients were analyzed, as summarized in Table 1. The risk of bias was assessed individually for each included study, with particular attention to study design and reporting quality. For every analysis, the characteristics of the included studies and their risk of bias were analyzed to contextualize the strength and reliability of the evidence. Differences in study populations, interventions, and outcome measures were evaluated to identify potential sources of heterogeneity. The studied group consisted of 23 females and 11 males. The average age at symptom onset was 46.85 ± 13.08 years, and the average age at the diagnosis of hMF was 50.79 ± 12.93 years. One report did not state the precise age but described the patient as being in their late 50s at the time of diagnosis, and the duration of lesions was reported to be about eight years [6]. This case clearly met the inclusion criteria of the review; however, it was not included in the analysis of the average age at symptom onset and diagnosis. In the analyzed group, 23 patients were in stage IA at the moment of diagnosis, 7 in stage IB, and for 4 patients, staging was not assessed. CD4/CD8 staining was reported in 12 cases, and among them, 7 were CD4+ and 5 were CD8+. T-cell receptor gene rearrangement was reported in only four cases (Case 2—positive, Case 15—negative, Case 16—positive, and Case 33—positive) [5,6,7,17].

As for phototype and ethnicity, these characteristics were not reported consistently across the articles. Only two authors used the Fitzpatrick scale [14,15]. Moreover, for cases 15, 16, and 17, the phototype was not stated in the text but could be inferred from the patients’ photographs as Fitzpatrick type IV/V [6,17,21]. Given this inconsistency, comparison between phototypes was not possible.

In order to compare treatment effectiveness, patients were divided into groups based on the treatment modalities: PUVA (psoralen plus ultraviolet A) with or without additional topical therapy; UVB (ultraviolet B) with or without additional topical treatment; and other treatment methods. A total of 2 patients received both UVB and PUVA therapy [15], and each course of treatment was analyzed separately. One patient received UVA1 therapy after achieving partial remission with UVB [15]. However, since this was the only case treated with UVA1, this modality of phototherapy was not analyzed separately. In this dataset, the choice between PUVA and UVB was not randomized and occurred within a heterogeneous clinical context, where treatment decisions were likely shaped by disease severity or physician preference. Any apparent differences in treatment outcomes should therefore be viewed cautiously, as they reflect observational evidence. In addition, the lack of some patients’ characteristics and missing critical information led to the exclusion of some cases of adult-onset hMF. A comparison of PUVA and UVB phototherapy is summarized in Table 2.

Among 17 patients treated with PUVA, 13 cases achieved complete remission (CR) and 3 cases achieved partial remission (PR). One patient’s outcome was defined in the article as ‘alive with disease’ [7]. Out of sixteen cases with reported remission, four of them experienced relapse (25%): three patients in a complete remission group, and one patient with partial remission. The longest-lasting complete remission was 120 months.

Fifteen patients were treated with UVB, and among them, one patient received two courses of UVB phototherapy [19], which were analyzed separately. Relapses were experienced in half of the UVB courses. A total of 5 out of 8 patients with CR, and 3 out of 8 patients with PR, relapsed. The longest-lasting complete remission was 72 months.

Among the analyzed cases, three showed progressive disease [12,13]. Sigal et al. [12] reported a 64-year-old white female with mixed hypopigmented and erythematous lesions accompanied by pruritus. At initial presentation, lymph node involvement was confirmed, with no involvement of the blood or internal organs. Immunostaining revealed a CD4+ dominant infiltrate in the skin sample. The patient was initially treated with topical mechlorethamine and etretinate, achieving partial repigmentation and reduced lesion infiltration, but the lymph nodes remained unaffected. Over several months, the disease progressed to the tumoral stage. Chemotherapy with cyclophosphamide, vincristine, and prednisone was ineffective, while total beam electron therapy resolved the cutaneous tumors, with no effect on lymph nodes. The patient died within two years from septicemia and bone marrow aplasia [12].

In the series reported by Amorim et al. [13], a 47-year-old female of mixed origin presented initially with a 2-year history of hypochromic macules on the trunk and extremities. The disease was staged at IB (T2aN0M0). Unfortunately, PUVA therapy was only partially effective, and after 2 years, she progressed to stage IIB (T3N1M0). Chemotherapy with gemcitabine was unsuccessful, and bone marrow became infiltrated by lymphoma. The patient died four years after the diagnosis. Another reported case in the same series, of a 68-year-old male of black origin who had complete remission after PUVA, experienced a recurrence of hMF after 8 years. He was referred to the hematology department due to advanced disease at stage IIIA (T4N1M0), and unfortunately was lost to follow-up [13].

## 4. Discussion

### 4.1. Diagnosis of Hypopigmented Mycosis Fungoides

The diagnosis of mycosis fungoides is established through a combination of clinical, histopathological, immunophenotypic, and molecular findings. In early-stage and rare variants of MF, skin lesions can mimic many dermatoses, and thus, diagnosis often requires multiple biopsies over time. The histopathologic hallmark of classic mycosis fungoides is epidermotropism of atypical T-lymphocytes without significant spongiosis. Atypical T-cells are small-to-medium-sized with cerebriform nuclei. The presence of Pautrier microabscesses is highly specific for MF, but their sensitivity is low, as they are observed in only a minority of cases [22].

Compared with classic MF, epidermotropism in hMF occurs with similar frequency. However, Pautrier microabscesses are significantly less common. In addition, the dermal infiltrate and dermal atypia are less pronounced, making the diagnosis of hMF even more challenging [23,24]. In inconclusive cases, basement membrane thickness may aid in diagnosis. In a study conducted by Prof. Abdelkader [25], a cut-off value of 32.7 μm was identified, yielding a sensitivity of 85.7% and a specificity of 96% for hMF. The author proposed a histopathologic criterion for hMF of basement membrane thickness exceeding 33 μm [25].

Immunophenotype of T-cells differs between hMF and non-hMF. While in hMF, the infiltrate consists of usually CD8+ cells, and a classic MF CD4+ dominant immunoprofile is observed [2,22,23]. Notably, CD4+, CD4/CD8 double-positive and double-negative cases of hMF were also reported in the literature [3,26,27]. T-cell receptor gene rearrangement may demonstrate mono- or polyclonality and assist diagnosis in difficult cases [28]; however, in the analyzed articles, it was not commonly utilized. Another promising marker is thymocyte selection-associated high-mobility group box (TOX). Its increased expression was demonstrated in hMF, with a proposed cut-off value of 1.5 providing 93.3% sensitivity and specificity [29]. However, cases of hMF lacking TOX expression have been reported, indicating that this biomarker requires further validation [30].

Non-invasive imaging techniques may aid in the early detection of hMF. Reflectance confocal microscopy (RCM) can be used to differentiate vitiligo from hMF lesions. According to a Chinese study [31], vitiligo lesions showed marked melanin reduction or its complete loss, sometimes accompanied by mild superficial dermal inflammation. In contrast, hMF lesions exhibited only slight melanin reduction and characteristic RCM features: weakly refractile round cells in vesicle-like epidermal spaces (corresponding to Pautrier microabscesses) and scattered cells in the papillary dermis. Importantly, these findings were confirmed by histopathological examination [31]. Dermoscopy may also be useful in the diagnostic work-up of hypopigmented MF as well. The most common dermoscopic features include patchy, amorphous white-pink areas with ill-defined borders, a weak or lost natural pigment network, and spermatozoa-like blood vessels [32,33]. These additional clues may help to distinguish hMF from other hypopigmented dermatoses.

In summary, although hypopigmented mycosis fungoides is primarily diagnosed through integrated clinical, pathological, and immunophenotypic assessment, emerging imaging techniques and biomarkers can enhance accuracy when conventional evaluations are insufficient.

### 4.2. The Role of CD8+ T-Cells

Although predominant CD8+ cell epidermotropism is considered a hallmark of hypopigmented mycosis fungoides [2], emerging evidence challenges this paradigm [34]. The exact role of CD8+ T-cells in the pathogenesis of hMF remains unclear. These neoplastic cells are thought to damage melanocytes by decreasing CD117 expression and the secretion of microphthalmia-associated transcription factor [35,36,37]. The interplay between these two molecules is critical for regulating melanin production, and changes in their expression result in the formation of hypopigmented skin lesions. While this process accounts for the hypochromic appearance of hMF lesions, CD8+ predominance is traditionally also believed to underlie its more indolent clinical course. According to some studies, the cytokine profile characteristic of CD8+ cells may create a tumor-suppressive microenvironment that restricts the expansion of the neoplastic clone [38].

In some patients, the epidermotropic CD8+ T-cells were found to have clonal T-cell receptor rearrangements, confirming that they represented the malignant T-cell clone [7]. However, recent findings suggest that the abundant CD8+ cells in hMF may represent a reactive infiltrate rather than the malignant clone, and that pathogenic CD4+ cells may be present at low levels, potentially below the detection threshold of current routine methods [34]. This questions the assumption that hMF is mediated by CD8+ cells and highlights the need for further studies exploring its immunophenotype and origin. Clearly, further studies are needed to better understand the mechanism behind hMF.

### 4.3. Hypopigmented Mycosis Fungoides vs. Mycosis Fungoides with Hypopigmented Lesions

Hypopigmented lesions in patients with mycosis fungoides may sometimes be challenging. They can develop as primary lesions in the hypopigmented variant of mycosis fungoides or may develop later, in the course of classic mycosis fungoides. The distinction between these two variants carries significant implications and is crucial in determining correct diagnosis, prognosis, and treatment approach.

Leukoderma in primary hypopigmented mycosis fungoides has traditionally been attributed to the cytotoxic effects of a CD8+ T-cell infiltrate directed against melanocytes [35,36,37]. However, in light of recent data suggesting that CD8+ cells could represent a reactive immune response, hypopigmentation may reflect effective local immune surveillance rather than a distinct pathogenic mechanism [34]. Although the exact mechanism underlying hypopigmentation in hMF remains incompletely understood, patients with this variant usually respond well to skin-directed therapies and require less aggressive treatment decisions. Early-stage hMF can be successfully managed with topical corticosteroids and phototherapy in primary treatment, as well as in recurrences [2].

Patients with secondary hypopigmentation in the course of MF tend to have a classic CD4+ T-cell infiltrate. Hypopigmented lesions might reflect increased melanocyte-directed cytotoxicity of clonal or reactive T-cells, or post-inflammatory pigment changes. Sometimes, it may be considered a marker of good prognosis [39]. Nonetheless, in every case, hypochromia warrants closer monitoring for signs of progression, especially if it is associated with plaques and tumor formation. Then, it indicates disease evolution and requires more aggressive treatment. Anytime vitiligo-like lesions develop in the course of MF, careful follow-up should be implemented. Bouloc et al. [40] presented a series of four patients with CTCL, who developed hypochromic lesions during the flares of pre-existing Sezary syndrome and erythrodermic MF. All presented cases had CD8-negative infiltrate. Hipochromic lesions developed on average 3.5 years after the onset of erythematous lesions and regressed with the improvement of the disease [40]. Similar reports of generalized leukoderma in the course of Sezary syndrome were described in the literature [41,42], as well as the occurrence of vitiligo-like leukoderma during phototherapy in patients with MF [43,44].

The authors highlight the necessity of distinguishing these conditions from true hMF, given the substantial differences in prognosis and the need for tailored therapeutic approaches. A concise summary of the distinguishing features between hypopigmented mycosis fungoides and mycosis fungoides with hypopigmented lesions is provided in Table 3.

### 4.4. Hypopigmented Mycosis Fungoides vs. Hypopigmented T-Cell Dyscrasia

Although clinically, lesions in the course of hypopigmented T-cell dyscrasia (HTCD) and hypopigmented mycosis fungoides may be indistinguishable, it is important to differentiate between them. HTCD is a variant of T-cell dyscrasia presenting with hypopigmented skin lesions and is regarded as a form of large-plaque parapsoriasis (LPP) [45]. Both conditions present with hypopigmented lesions in photo-protected areas, involve clonal T-cell proliferation, and share histopathological features, including small-to-medium-sized lymphocytes with cerebriform nuclei, lymphoid atypia, and epidermotropism [45]. They share histopathological findings as well, such as small- and medium-sized lymphocytes with cerebriform nuclei, lymphoid atypia, and epidermotropism. Distinguishing features of hMF include a denser lymphocytic infiltrate, Pautrier microabscesses, and folliculotropism [45,46].

Immunohistochemically, both conditions often show CD8+ predominance, but hMF demonstrates a more pronounced reduction in CD7 and CD62L expression [45]. Therapeutic management primarily relies on skin-directed approaches, including topical corticosteroids and UVB phototherapy for both disorders, with PUVA preferentially used in hMF. Evidence indicates that UVB rapidly reduces epidermal lymphocytic infiltration but has limited effects on dermal lymphocytes. Consequently, some authors advise against UVB use in HTCD/hMF or, when PUVA is contraindicated, continuing treatment until histopathologic remission is achieved before cessation [47].

There is an ongoing debate in the literature. While some researchers consider LPP, HTCD, and hMF to be stages of the same process [45,48,49], others emphasize that progression from HTCD to hMF appears to be exceedingly rare [46]. Illustrating the spectrum concept, Chuang et al. [48] reported a case of a 61-year-old African American female with a 20-year history of pruritic, non-scaly hypopigmented patches distributed over her entire body. Early skin biopsies were inconclusive, leading to a diagnosis of T-cell dyscrasia. Upon receiving etanercept for arthritis, her lesions worsened over six months. Subsequent biopsies confirmed cutaneous T-cell lymphoma. After discontinuing the biologic therapy, the patient’s lesions improved with methotrexate and phototherapy [48]. In this patient, etanercept may have acted as a trigger for disease progression from HTCD to hMF. Accordingly, the use of biologic agents, particularly TNF-α inhibitors, should be approached with caution or avoided in individuals with a history of lymphoma, T-cell dyscrasia, or other T-cell-mediated disorders.

Another noteworthy case involving hypopigmented lesions in the setting of biologic therapy was reported by Balboul et al. [50]. They reported a young female patient developing an hMF-like eruption 20 weeks after initiating ixekizumab for psoriasis. Histopathologic examination revealed epidermotropism of atypical lymphocytes with CD8+ predominance and TCR monoclonality. Clinical improvement was observed after ixekizumab was discontinued and topical corticosteroids were introduced [50].

Current evidence suggests that biologic therapies do not directly cause mycosis fungoides, but may contribute to the clinical emergence of latent or early disease [51]. Therefore, caution and careful monitoring are warranted when new or atypical skin lesions develop during biological treatment.

### 4.5. Treatment of Hypopigmented Mycosis Fungoides

Treatment for hMF is primarily skin-directed, with excellent response rates. Phototherapy is the cornerstone of management: narrowband UVB is preferable in children, while PUVA may be used in adolescents or adults. UVA1 is less frequently used due to limited availability. Compared with classic MF, response rates are generally higher in hMF [52]. Our analysis indicates that PUVA therapy may be superior to UVB, with patients achieving complete remission more frequently, experiencing fewer relapses, and enjoying longer-lasting remissions. These findings are supported by data from the literature [52]. This may be explained by the greater depth of UVA penetration and the enhanced cytotoxic effects of psoralen-mediated photochemotherapy. Psoralen intercalates between DNA strands and, after activation by UVA radiation, forms thymine cross-links. These DNA–psoralen cross-bonds prevent normal DNA replication, leading to cell-cycle arrest and cell death. Moreover, UVA is less absorbed by melanin compared with UVB, potentially leading to improved efficacy in darker-phototype populations [53]. To enhance the efficacy of UVB therapy, combining it with topical corticosteroids may be considered [54]. For solitary lesions, a targeted approach with excimer laser has been shown to produce excellent results while avoiding the risks associated with broader phototherapy [55,56]. In cases of limited disease, topical treatments such as nitrogen mustard or corticosteroids can also be effective [57].

Systemic therapy is typically reserved for refractory or progressive hMF, including low-dose methotrexate, systemic retinoids, or radiotherapy. Total beam electron therapy and chemotherapy are used only in very advanced cases [13]. For patients experiencing severe pruritus, aprepitant may be considered as a symptomatic treatment [58]. In our practice, we encountered one case of refractory hMF: an adult-onset male patient who did not respond to topical or systemic corticosteroids combined with PUVA therapy. He was subsequently treated with a combination of methotrexate and mogamulizumab, achieving partial remission [59].

Differences in treatment approach between children and adults reflect safety considerations rather than disease behavior. In children, UVB phototherapy is the preferred first-line treatment because of its favorable safety profile, excellent efficacy, and the absence of psoralen, which is contraindicated in children under 12 years of age. Topical therapy may be sufficient in limited disease, and systemic therapy is rarely necessary [60,61,62,63,64,65].

Although hMF responds well to skin-directed therapies, recurrences are common. One possible explanation is minimal residual disease (MRD), defined as the persistent presence of a malignant T-cell clone in the skin despite clinical remission [66]. Data on MRD in MF are limited, and its prognostic significance remains unclear. Hsiao et al. [67] reported a case of a young boy with stage IA hMF who was successfully treated with carmustine. One month after achieving clinical remission, a skin biopsy from the previously affected site revealed no histologic evidence of disease. However, TCR gene analysis detected persistence of the original malignant T-cell clone. Notably, at 3½ years of follow-up, the patient remained free of clinical relapse [67]. Currently, no formal guidelines recommend checking for MRD prior to stopping therapy, though it may be considered in multiple recurrences.

### 4.6. Prognosis of Hypopigmented Mycosis Fungoides

HMF is generally associated with an excellent prognosis in both children and adults, with studies indicating a 68% reduction in progression risk compared with other MF variants [68]. Direct comparative data specifically contrasting outcomes of hMF in children versus adults are limited. HMF often represents the predominant MF variant in pediatric series, especially in darker phototypes, whereas adult hMF is a minority of all MF cases. Pediatric-onset mycosis fungoides demonstrates a lower progression rate compared with adult-onset disease, supporting its more indolent course [69]. Both pediatric and adult hMF often respond well to phototherapy, although recurrence rates are high, and long-term follow-up is necessary [70].

In a cohort of patients, individuals with hMF demonstrated a 10-year survival rate of 100%, compared with 51.2% in those without hypopigmentation [68]. Progression in the course of mycosis fungoides is associated with a more advanced stage at diagnosis, lymph nodes and blood involvement, TCR clonality in the blood, large cell transformation, and elevated lactate dehydrogenase [68]. Disease progression in hMF generally affects patients who initially present with advanced-stage disease or in patients who miss scheduled follow-up visits [13,71]. Large cell transformation is highly unlikely, although still possible [72,73,74]. Furthermore, cases of hMF have been reported in association with other medical conditions [6,75,76,77,78]. Although recurrences are common, they do not appear to confer a worse overall prognosis [2]. Notably, the predominance of CD8+ T-cells has been proposed by many authors as a factor contributing to less aggressive behavior of these subtypes of MF [2,7,79,80]. It has even been suggested that aggressive cases labeled as CD8+ MF in earlier literature may actually represent misdiagnosed cases of primary cutaneous aggressive epidermotropic CD8+ T-cell lymphoma [81].

Although there is a generally favorable outlook for hMF, long-term outcomes remain incompletely determined. Only a limited number of cases with extended follow-up have been reported in the literature [3,13]. In our cohort, the longest follow-up was 12 years. Careful long-term monitoring is necessary, as relapses may develop despite complete clinical remission.

### 4.7. Limitations

A major limitation of this review is that the evidence consists almost entirely of individual case reports and small case series, which represent the lowest level of clinical evidence. Treatment outcomes reported for PUVA and UVB cannot be directly compared in a robust way, as the underlying data was not drawn through a controlled study design. The choice of therapy may have been influenced by patient-specific factors, such as disease severity, prior treatment history, or clinician preference, which were not standardized. Accordingly, the results should be regarded as exploratory and hypothesis-generating rather than conclusive. The lack of larger prospective or randomized studies restricts both the strength and applicability of these observations, highlighting an urgent need for prospective studies. As such, any apparent advantage of PUVA over UVB should be considered preliminary, and these results serve only to generate hypotheses rather than to provide definitive guidance.

Moreover, patients’ characteristics, e.g., phototype, were not reported consistently across cases, and missing information on clinical course, treatment response, or follow-up led to the exclusion of some cases of adult-onset hMF. This limitation both affects case inclusion and illustrates the importance of thorough, standardized reporting in clinical practice.

## 5. Conclusions

Hypopigmented mycosis fungoides predominantly affects children and young adults, but it should also be included in the differential diagnosis of hypopigmented lesions in adults over 30. Diagnosis can be challenging, as histopathological findings are not always definitive, and repeated biopsies may be necessary. Hypopigmented T-cell dyscrasia should be considered when findings are inconclusive. In adults with early-stage hMF, PUVA therapy is generally recommended, though treatment regimens are not standardized, and the decision to discontinue therapy should ideally rely on histopathologic confirmation rather than clinical improvement alone. Prognosis is favorable, yet recurrences are common, highlighting the need for regular follow-up.

## Figures and Tables

**Table 1:** The summary of analyzed cases. Cauc.—Caucasian, AA—African American, N.A.I.—Native American Indian, Nicar.—Nicaraguan, m—male, f—female, CR—complete remission, PR—partial remission, AD—alive with disease, PD—progressive disease, tGCS—topical glicocostricosteroids, UVB—ultraviolet B phototherapy, UVA1—ultraviolet A1 phototherapy, PUVA—psoralen plus UVA photochemotherapy, BCNU—topical carmustine, HN2—mechlorethamine, CVP—chemotherapy with cyclophosphamide, vincristine, and prednisone, Gem—chemotherapy with gemcitabine, and ND—no data.

Case No.	Phototype/Ethnicity	Sex	Age at Symptom Onset (Years)	Age at Diagnosis (Years)	Staging	CD4/CD8	Treatment	Outcome	Follow-Up (Months)	Final Outcome
1 [16]	White	f	31	32	IA	CD4+	PUVA	CR	12	CR
2 [7]	White	f	43	44	IA (T1N0M0)	CD8+	tGCS + sunlight	AD	12	AD
3 [7]	White	f	41	42	IB (T2N0M0)	ND	PUVA	AD	12	AD
4 [18]	Cauc.	f	39	39	ND	CD8+	UVB	PR	6	relapse
							tGCS	PR	12	PR
5 [13]	Cauc.	m	38	53	IB (T2bN0M0)	ND	tGCS + PUVA	CR	120	CR
6 [13]	Cauc.	f	53	54	IA (T1aN0M0)	ND	tGCS + UVB	PR	144	PR
7 [12]	Cauc.	f	64	64	ND	CD4+	HN2 + etretinate	PD	22	death
							CVP	PD		
							total beam electron	death		
8 [13]	Cauc.	m	62	63	IA (T1aN0M0)	ND	tGCS + PUVA	PR	96	PR
9 [13]	Cauc.	f	57	63	IA (T1bN0M0)	ND	tGCS + PUVA	CR	96	CR
10 [15]	III	f	61	61	IA	ND	UVB	CR	7	relapse, with good response to UVB
11 [15]	IV	m	34	34	IA	ND	UVB	PR	ND	ND
							UVA1	CR	108	CR
12 [15]	IV	f	31	39	IB	ND	UVB	CR	2	relapse
							PUVA	CR	ND	ND
13 [15]	IV	f	36	41	IA	ND	UVB	PR	4	relapse, with good response to UVB
14 [15]	IV	f	36	37	IA	ND	UVB	CR	7	relapse, with good response to tGCS
15 [17]	IV–V	m	30	50	IA	CD8+	UVB	CR	12	CR
16 [6]	IV–V	f	40/50s	50s	IA	CD4+	UVB	CR	12	CR
17 [21]	IV–V	f	30	31	IA (T1b)	CD4+	tGCS + UVB	PR	4	PR
18 [14]	V	f	51	55	IA	ND	UVB	CR	1.5	relapse
19 [14]	V	f	52	52	IA	ND	UVB	PR	1.5	relapse
20 [15]	V	m	60	60	IA	ND	UVB	PR	ND	ND
							PUVA	CR	2	CR
21 [20]	AA	m	33	39	IA	CD4+	PUVA	CR	36	relapse with good response to PUVA
22 [20]	AA	m	30	40	IA	CD4+	PUVA	CR	5.5	CR
23 [20]	AA	m	45	46	IA	CD4+	PUVA	CR	24	relapse with good response to PUVA
24 [19]	AA	f	49	74	ND	CD8+	UVB	CR	6	relapse
							UVB	PR	2	PR
25 [13]	Black	m	40	41	IB (T2aN0M0)	ND	tGCS + UVB	CR	72	CR
26 [13]	Black	f	61	69	IB (T2bN0M0)	ND	PUVA	CR	72	CR
27 [13]	Black	f	44	48	IA (T1aN0M0)	ND	tGCS + PUVA	CR	84	CR
28 [13]	Black	m	67	68	IA (T1aN0M0)	ND	PUVA	CR	96	relapse
29 [13]	Mixed	f	45	47	IB (T2aN0M0)	ND	PUVA	PR	24	PD
							Gem	PD	24	death
30 [13]	Mixed	f	75	75	IA (T1bN0M0	ND	tGCS + PUVA	CR	60	CR
31 [13]	Mixed	f	59	60	IB (T2bN0M0)	ND	PUVA	CR	72	CR
32 [13]	Mixed	m	34	35	IA (T1aN0M0)	ND	PUVA	PR	84	PR
33 [7]	N.A.I.	f	43	45	IA (T1N0M0)	CD8+	BCNU + tGCS	AD	24	AD
34 [5]	Nicar.	f	72	75	ND	ND	HN2	PR	2	PR

**Table 2:** Comparison of PUVA and UVB phototherapy effectiveness.

	PUVA	UVB
No. of treatment courses	17	16
Complete remission	13	8
Partial remission	3	8
Longest-lasting complete remission (months)	120	72
Relapse after remission (complete or partial)	4 (25%)	8 (50%)

**Table 3:** Comparison of hypopigmented mycosis fungoides vs. mycosis fungoides with hypopigmented lesions [2,35,36,37,39,40].

Feature	Hypopigmented Mycosis Fungoides	Mycosis Fungoides with Secondary Hypopigmented Lesions
Clinical presentation	Predominantly hypopigmented patches or plaques	Typical MF lesions (patches, plaques, and nodules) with hypopigmentation developing secondarily
Histopathology	Subtle epidermotropism, minimal epidermal damage	Typical MF histology
Immunophenotype	In most cases, CD8+ predominant	In most cases, CD4+ predominant
Patomechanism	Immune-mediated melanocyte damage	Secondary melanocyte dysfunction due to inflammation or treatment; in rare cases, disease progression
Prognosis	Generally favorable, indolent course	Usually associated with good prognosis; occasionally linked to progression

## Abbreviations

The following abbreviations are used in this manuscript:

MF	Mycosis fungoides
hMF	Hypopigmented mycosis fungoides
CTCL	Primary cutaneous T-cell lymphoma
TCR	T-cell receptor
UVB	Ultraviolet B
PUVA	Psoralen plus ultraviolet A
UVA1	Ultraviolet A1
TOX	Thymocyte selection-associated high-mobility group box
RCM	Reflectance confocal microscopy
HTCD	Hypopigmented T-cell dyscrasia
LPP	Large-plaque parapsoriasis
MRD	Minimal residual disease
